# T2SS-peptidase XcpA associated with LasR evolutional phenotypic variations provides a fitness advantage to *Pseudomonas aeruginosa* PAO1

**DOI:** 10.3389/fmicb.2023.1256785

**Published:** 2023-10-26

**Authors:** Mengmeng Cheng, Ruiyi Chen, Lisheng Liao

**Affiliations:** ^1^Guangdong Province Key Laboratory of Microbial Signals and Disease Control, Integrative Microbiology Research Centre, South China Agricultural University, Guangzhou, China; ^2^Department of Microbiology, University of Washington, Seattle, WA, United States

**Keywords:** microevolution, bacterial evolution, virulence factors, *Pseudomonas aeruginosa*, quorum sensing, pathogenicity traits

## Abstract

The Gram-negative opportunistic pathogen *Pseudomonas aeruginosa* possesses hierarchical quorum sensing (QS) systems. The intricate QS network of *P. aeruginosa* synchronizes a suite of virulence factors, contributing to the mortality and morbidity linked to the pathogenicity of this bacterium. Previous studies have revealed that variations in the *lasR* gene are frequently observed in chronic isolates of cystic fibrosis (CF). Specifically, LasR^Q45stop^ was identified as a common variant among CF, *lasR* mutants during statistical analysis of the clinical *lasR* mutants in the database. In this study, we introduced LasR^Q45stop^ into the chromosome of *P. aeruginosa* PAO1 through allelic replacement. The social traits of PAO1 LasR^Q45stop^ were found to be equivalent to those of PAO1 LasR-null isolates. By co-evolving with the wild-type in caseinate broth, elastase-phenotypic-variability variants were derived from the LasR^Q45stop^ subpopulation. Upon further examination of four LasR^Q45stop^ sublines, we determined that the variation of T2SS-peptidase *xcpA* and *mexT* genes plays a pivotal role in the divergence of various phenotypes, including public goods elastase secretion and other pathogenicity traits. Furthermore, XcpA mutants demonstrated a fitness advantage compared to parent strains during co-evolution. Numerous phenotypic variations were associated with subline-specific genetic alterations. Collectively, these findings suggest that even within the same parental subline, there is ongoing microevolution of individual mutational trajectory diversity during adaptation.

## Introduction

*Pseudomonas aeruginosa* is an opportunistic bacterial pathogen responsible for acute and chronic lung infections in immune-compromised individuals, including cystic fibrosis (CF) patients. This leads to high mortality and morbidity rates (Lyczak et al., 2000; Ahmed et al., 2019). The bacterium poses a significant public health challenge due to its extensive metabolic versatility, swift adaptation to diverse stresses, innate resistance, tendency to develop antibiotic resistance, biofilm formation capacity, and virulence factor secretion. Quorum sensing (QS), predominantly governs these traits, making eradication notably difficult (Huang et al., 2019; Lu et al., 2022).

*P. aeruginosa* features two primary acyl-homoserine lactone (AHL) QS circuits: LasIR and RhlIR (Tashiro et al., 2013). Specifically, the signal synthase LasI synthesizes 3-oxo-dodecanoyl-homoserine lactone (3OC12HSL), which the QS regulator LasR receives, activating the transcription of numerous genes. The LasR QS regulon includes over 300 genes, like *lasI*, *rhlI*, and *rhlR*. In contrast, the secondary QS regulator RhlR detects N-butanoyl-HSL (C4-HSL) produced by signal synthase RhlI. The RhlR regulon covers various genes, some of which overlap with the LasR regulon (Schuster et al., 2013). Genes regulated by LasR and RhlR encode essential secreted virulence factors, including protease elastase, hydrogen cyanide, and phenazines (Qin et al., 2022). The *Pseudomonas* quinolone signal (PQS) system, another non-AHL QS system in *P. aeruginosa*, involves the transcriptional regulator PqsR (also termed MvfR). This binds to 2-heptyl-3-hydroxy-4-quinolone (PQS) or its biosynthetic precursor, 2-heptyl-4-quinolone (HHQ), produced by PqsABCD and PqsH, respectively (Xiao et al., 2006).

Through genetic mutations and modifications in its quorum sensing regulatory genes, *P. aeruginosa* can induce phenotypic changes, preparing the bacterium for diverse environments, including the CF lung (Chugani et al., 2012; Folkesson et al., 2012). One such adaptation in chronic CF isolates is the mutational inactivation of the *lasR* gene. Often, LasR loss-of-function mutations trigger AHL and LasB-negative phenotypes in chronic *P. aeruginosa* CF isolates (Seed et al., 1995; Kiratisin et al., 2002; Wang et al., 2018). When QS is vital for growth in the *P. aeruginosa* PAO1 background, LasR mutants gain a fitness advantage due to reduced metabolic costs when co-cultured with wild-type *P. aeruginosa* PAO1. This wild-type provides shared public goods for the entire cell population (Schuster et al., 2003). Yet, if the LasR mutant population surpasses a certain threshold, it collapses (Wang et al., 2015). Introducing C4-HSL or co-evolving bacteria that produce C4-HSL with PAO1 *lasR* mutants swiftly leads to populations with an active RhlIR QS system (Kostylev et al., 2019). Many *lasR* mutant clinical isolates still respond to AHL via the RhlRI system (Chen et al., 2019; Cruz et al., 2020). Today, advancements in high-throughput DNA sequencing technologies allow millions of DNA sequences from a single microbial sample. This technology has evolved into an efficient tool for determining genetic adaptations in infected host populations (Kostylev et al., 2019).

Previous studies noted that LasR locus analysis of *P. aeruginosa* isolates from CF patients identified LasR^Q45stop^ as a frequent mutation site (Cruz et al., 2020). This research aims to explore the evolutionary pathways in lab conditions focusing on the high-frequency mutation site of the LasR^Q45stop^ variation, which has a single substitution at base 133 in *lasR*. Laboratory-induced mutations of LasR^Q45stop^ in strain PAO1 indicate it as a nonfunctional LasR. When co-cultured in casein broth with cooperator PAO1, LasR^Q45stop^ spurred the swift emergence of proteolysis-associated mutant variants. Multiple variants displayed characteristics similar to CF clinical isolates, including a functional RhlI-RhlR system due to a *mexT* mutation. Our study identified the *xcpA* mutant exhibiting numerous virulence-related attributes, including reduced extracellular protein production and twitching motility. Additionally, we present lab experiments elucidating why *mexT* and *xcpA* are prevalent in the context of LasR^Q45stop^ evolution.

## Materials and methods

### Bacterial strains, media, and growth conditions

Bacterial strains and plasmids used are described in the Supplementary Table S1. *P. aeruginosa* was cultivated in Luria-Bertani [0.5% (w/v) yeast extract, 1% (w/v) tryptone, and 1% (w/v) sodium chloride] broth with a pH of 6.8, using 50 mM 3-(N-morpholino)propanesulfonic acid as a buffer (LB-Mops broth), PM broth with an addition of 1% sodium caseinate (casein broth) (Kostylev et al., 2019), or *Pseudomonas* P broth [0.14% (w/v) magnesium chloride, 2% (w/v) pancreatic digest of gelatin, and 1% (w/v) potassium sulfate]. Several types of agar were used for plating purposes, including LB agar, and skim milk agar (Sandoz et al., 2007). Typically, 100 μg/mL of gentamicin, 50 μg/mL of kanamycin, or 300 μg/mL of carboxybenzylmycin was added to the medium. Bacteria were subcultured in 14 mm Falcon tubes with fresh LB or LB-Mops broth and incubated at 37°C with shaking at 250 rpm. *Escherichia coli* used in the cloning process was grown in LB broth.

### Strain construction

Our investigations utilized the *P. aeruginosa* PAO1 strain (Stover et al., 2000), which differs from the currently sequenced strain due to an 8 bp deletion at the *mexT* gene. Mutants with deletions of *lasR*, *lasR^Q45stop^*, *psdR*, *mexT*, *xcpA*, and *lasB* were derived by cloning PAO1 using the suicide vector pEXG2, as previously described (Hmelo et al., 2015). The Supplementary Table S1, lists the primers used to construct the pEXG2 knockout plasmids. Reverse-transcriptase PCR of genomic DNA confirmed all deletion mutations. To compensate for the mutations, we integrated wild-type copies of genes from pUC18-mini-Tn7-Gm, as previously reported (Choi et al., 2005).

### Assays for LasR activity, Cyanide and pyocyanin production

We measured promoter activity in cells cultured in LB-MOPS broth, as previously reported (Feltner et al., 2016), using *lasI-gfp* reporter strains in a 96-well plate. These were incubated at 37°C with continuous shaking in a Synergy H1 microplate reader (Biotec, America). GFP fluorescence (excitation at 485 nm, emission at 535 nm) and cell density (OD_600_) were recorded at 2 h intervals for 28 h (refer to Supplementary Table S1 in the Supplementary material). For the detection of cyanide, we employed a cyanogenic test paper approach detailed previously (Chen et al., 2019). Pyocyanin activity was estimated using the modified protocol of O’Loughlin et al. (2013). The *P. aeruginosa* PAO1 strain was cultured overnight and subsequently diluted to an OD_600_ of 0.01, followed by incubation in 3 mL of P broth medium for 24 h.

### Motility assays

This study assessed swarming motility using a medium composed of nutritional broth [0.8% (w/v)] supplemented with D-glucose [0.5% (w/v)] and solidified with 0.5% (w/v) Bacto Agar. Overnight liquid broth cultures were adjusted to an optical density of 1.0 before inoculation onto plates. Growth was evaluated after 18–24 h of incubation at 37°C. After 24 h of growth in LB medium containing 1% solidified Bacto Agar at 37°C, the agar was gently detached, revealing a twitching motility zone adhered to the petri dish. A 10 min stain with 1% (wt/vol) crystal violet made the twitching zones visible, and their diameters were subsequently measured (Liu et al., 2022).

### QS signal measurements

Quorum sensing signal extraction was carried out as described (Dong et al., 2008). The bacterium was cultivated in LB-Mops broth for 18 h, using 3 mL. Subsequently, an equal volume of acidified ethyl acetate (0.1% acetic acid) was combined with 3 mL of culture supernatant for signal extraction. Dried samples were reconstituted in 200 μL methanol and immediately filtered before LC-MS/MS analysis. We isolated and selected the prevalent signaling molecules for quantitative LC-MS/MS investigation. Modifications to the LC-MS procedure were made (Liu et al., 2022). HPLC was conducted using a Waters X Select HSS T3 1.8 μm phase column (2.1 × 100 mm) on the Dionex Ultimate 3000 system (Thermo Fisher Scientific). Metabolites identified included 3-oxo-C12-HSL (*m*/*z* 298.20128) and C4-HSL (*m*/*z* 172.09682). Data evaluation was executed with TraceFinder and Thermo Xcalibur, both from Thermo Fisher Scientific.

### Assessment of extracellular protease production

Bacteria grown on LB broth milk agar plates were incubated for 30 h at 37°C. The amount of extracellular protease produced was quantified by measuring the area of the proteolytic zone surrounding the bacterial colony.

### Competition experiments

Under these conditions, the gentamicin-resistance marker is neutral. We carried out competitive studies using *P. aeruginosa* strains both with and without this marker. The proportion of the competitor bearing the gentamicin-resistance marker was set at 1%, 10%, 50%, or 90% in casein broth for 48 h. To ascertain cell yields, plate counting, both with and without 10 μg/mL gentamicin, was employed to gauge proportional abundances after 48 h of incubation. The relative fitness was calculated by dividing the ratio of mutant to wild type after 48 h by the ratio at the time of inoculation.

### Social evolution experiments

For evolution experiments (Supplementary Figure S2C), log-phase LB-Mops cultures of both cooperator and competitor were prepared, adding the competitor to the casein broth at a ratio of 0.1 (total 100 μL). Two days post primary inoculation, once visible proliferation was observed, a 60 μL volume of the transfer inoculum was introduced daily into a 3 mL casein broth. Serial dilutions of isolates were grown on LB agar plates both with and without 10 μg/mL gentamicin to determine the percentage of protease-deficient cells. Cultures were sampled from LB agar plates containing 10 μg/mL gentamicin, and colonies were then streaked daily onto skim milk agar plates to detect protease-altering variations.

### Genome variant analysis

Bacteria were isolated from individual colonies and cultured in LB broth with a buffer for a duration of 24 h. Cells were collected for analysis via centrifugation. Bacteria were cultivated in LB-Mops broth, and genomic DNA was extracted using the Easy Pure Bacteria Genomic DNA Kit (TRAN). The genomic DNA of the four sublines (LasR^Q45stop^-1, LasR^Q45stop^-2, LasR^Q45stop^-3, and LasR^Q45stop^-4) was sequenced with the Illumina NovaSeq S4 PE-150 (Novogene, China). Whole Genomics Solution Corporation conducted the whole-genome sequencing and assembly. PCR testing was employed to validate the genomic sequencing results.

### Statistical analysis

Results are depicted as mean ± SD. Experimental groups were evaluated using an unpaired student’s *t*-test (one-tailed, unequal variance). Differences with a *p*-value <0.05 were considered significant. Experiments were repeated to confirm their consistency.

## Results

### The high-frequency mutant LasR^Q45stop^ presents fitness advantages over WT

**Figure 1:**
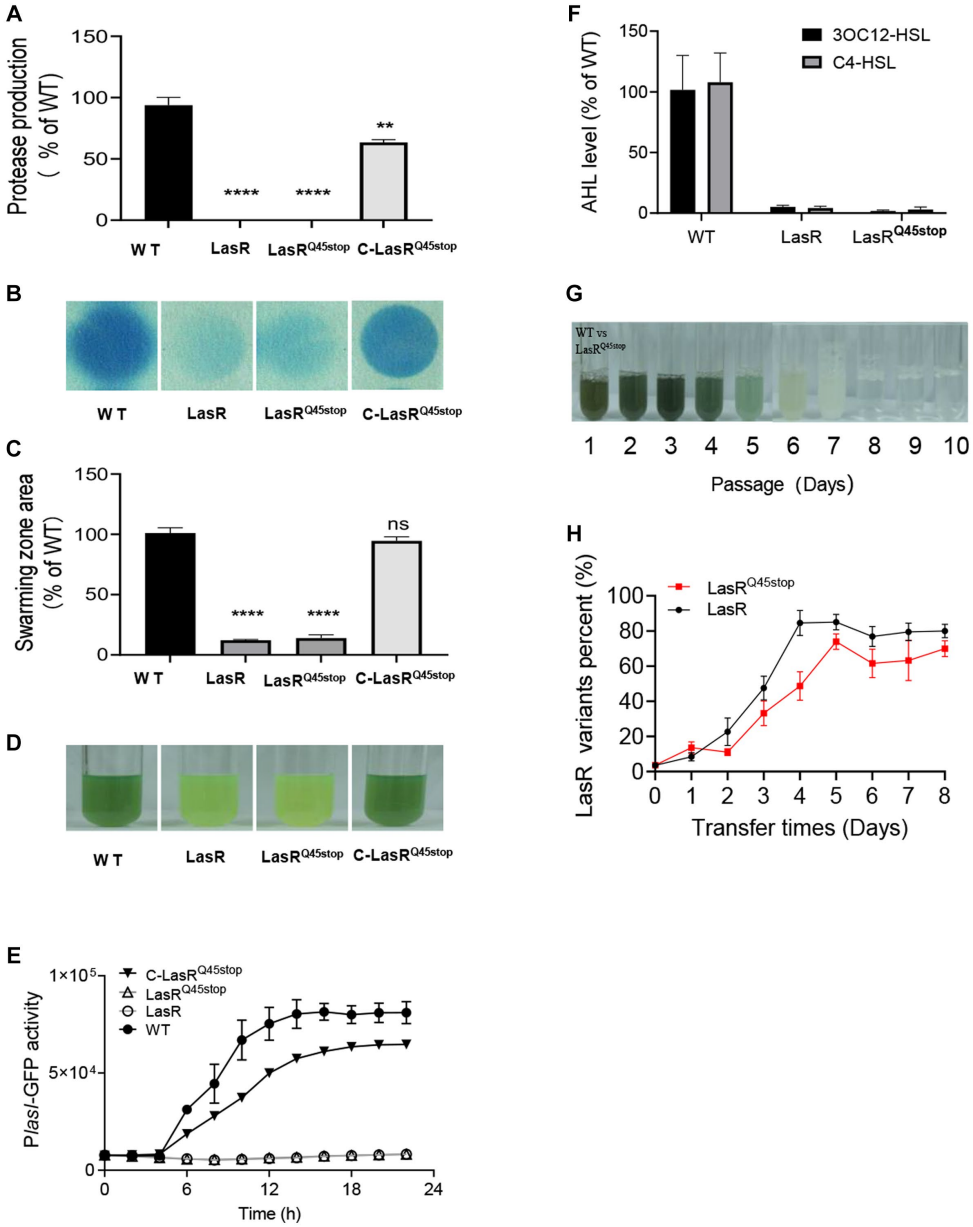
Characteristics in QS regulon of LasR^Q45stop^ in solitary presence and co-culture states. **(A)** Protease production on casein agar. The PAO1 LasR mutant and the LasR^Q45stop^ mutant produce relatively little protease. All data shown are the average values of three independent experiments ± SD. **(B)** Cyanide production by *P. aeruginosa* strains and mutants. Cyanide was monitored as described in Materials and methods. The blue coloration of the filter paper reflects cyanide production. **(C)** Swarming motility phenotype of PAO1 wild-type and LasR mutants. All data shown are the average values of three independent experiments ± SD. **(D)** Color changes of *P. aeruginosa* cultures in the presence of LasR mutant after 24 h, a reflection of the effect of LasR mutant on phenazine production. **(E)** LasR activity in the LasR mutant and PAO1 measured as GFP fluorescence (RFU). All strains contained the P*lasI*-gfp fusion plasmid pProbe-GT-P*lasI*gfp. **(F)** Relative AHL levels of the indicated strain. The AHL levels produced by strain PAO1 are defined as 100%. AHL was measured after 18 h of growth in LB-Mops broth. All data shown are the average values of three independent experiments ± SD. **(G)** An example of one serial transfer experiment. Each photographic image was captured after 1 day of growth. The relative abundance of LasR mutant (as a percentage of the total population) in culture samples just before transfer (60 μL) is distinguished by resistance markers, and the daily passage number is shown below the tubes. Cultures that only partially degrade casein have a milky white appearance due to protein precipitation. **(H)** Competition between WT PAO1 and LasR mutant (black), or LasR^Q45stop^ mutant (red). The initial frequency of LasR mutant was 0.01. Three biological replicates were performed. All data shown are the average values of three independent experiments ± SD. Two-tailed *p*-values were determined using unpaired *t*-tests. Statistical significance: ^*^p < 0.05, ^**^p < 0.01, ^***^p < 0.001, and ^****^p < 0.0001; ns, no significant difference (*p* ≥ 0.05).

The *lasR*^C133T^ substitution has been identified as a recurring mutation site in *P. aeruginosa* CF isolates (Feltner et al., 2016). This single-base substitution introduces a premature stop codon into the *lasR* sequence, terminating protein translation prematurely. This mutation was henceforth referred to as LasR^Q45stop^. To validate the functionality of LasR^Q45stop^, we generated a knock-out deletion mutant LasR, an allelic replacement mutant LasR^Q45stop^, and a LasR complementation strain C-LasR^Q45stop^ in the laboratory model strain *P. aeruginosa* PAO1. Comparing the QS-associated traits of the strains mentioned, we found that the homologous LasR^Q45stop^ mutation in *P. aeruginosa* acts as a nonfunctional LasR in QS-associated phenotypes, such as elastase secretion, pyocyanin production, and 3OC12-HSL and C4-HSL levels (Figures 1A–F).

To ascertain earlier studies indicated that LasR mutants possess fitness advantages over WT cells when co-cultured in minimal medium with casein (casein broth) as the sole carbon source. However, this advantage was negated with the addition of adenosine to the casein medium (Feng et al., 2019). To determine if LasR^Q45stop^ exhibited superior fitness to WT, we co-cultured the gentamicin-resistant PAO1 LasR^Q45stop^ mutant alongside WT in casein broth. LasR^Q45stop^ mutants were then distinguished and counted from WT using plates with or without gentamicin resistance. The schematic diagram (Supplementary Figure S2C) illustrates that 1% competitors (LasR^Q45stop^ or LasR) were individually combined with 99% of WT in the initial casein broth, then passaged daily, and the frequency of mutant strains was subsequently monitored. Pyocyanin production during the initial days of the evolution process hinted at the robust growth of the PAO1 co-culture. Pyocyanin levels tapered off from day 4 to day 6, culminating in a milky culture by day 7. No noticeable alteration in the casein medium post-day 8 signified a population collapse (Figure 1G). Consistent with expectations, the proportion of LasR^Q45stop^ mutants surged, peaking at nearly 70% in cultures (Figure 1H). This shift in frequency suggests that the LasR^Q45stop^ mutant can act as a social cheater when co-cultured with WT in a casein-based medium.

### Protease-change mutants rapidly emerge during growth of LasR^Q45stop^ in caseinate

**Figure 2:**
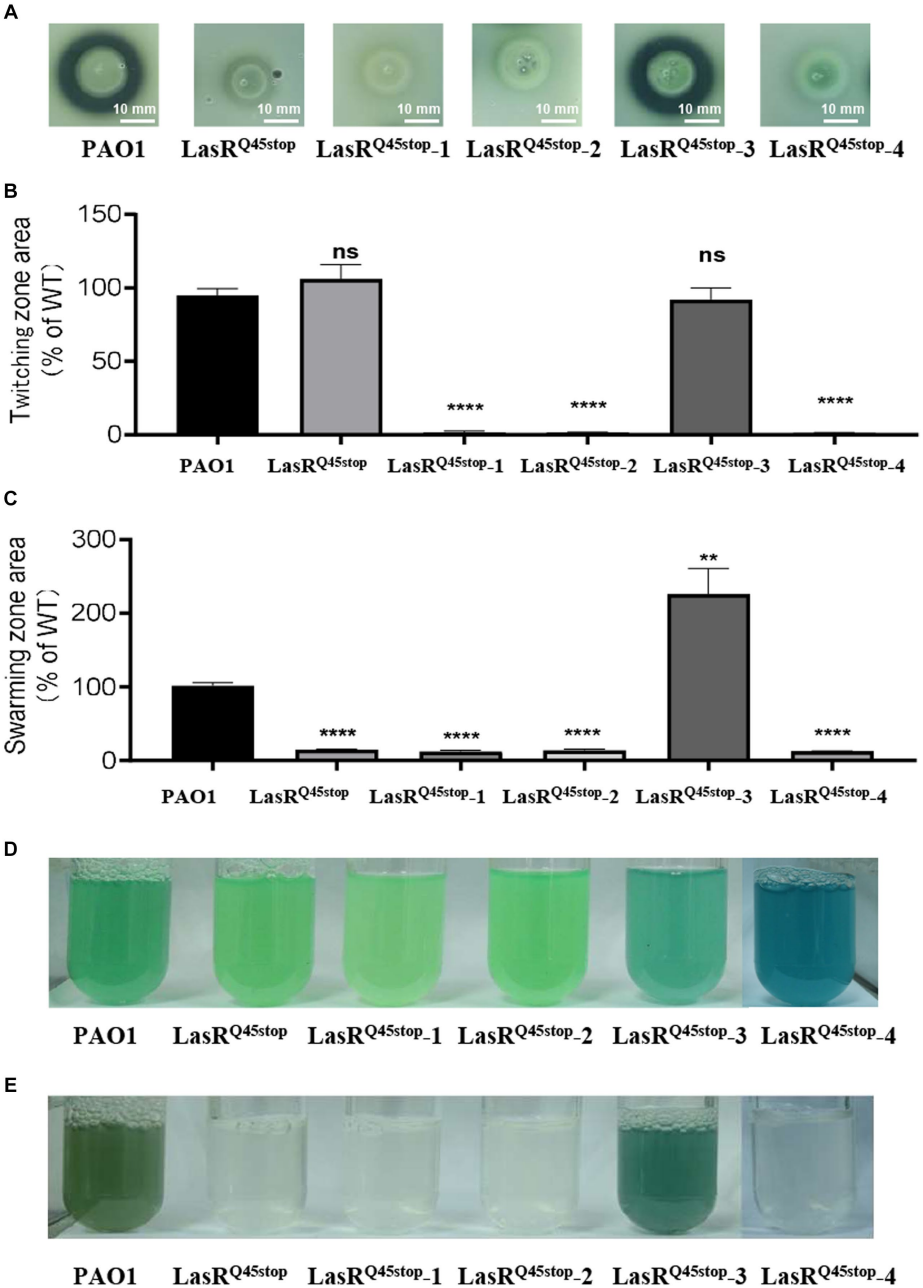
Characteristics of three protease-negative LasR^Q45stop^ mutant variants (LasR^Q45stop^-1, LasR^Q45stop^-2, and LasR^Q45stop^-4) and protease-producing LasR^Q45stop^ (LasR^Q45stop^-3) mutant variants derived from the LasR^Q45stop^ mutant. **(A)** Protease production of isolates from co-cultured casein broth was measured on plates containing 1% (*m*/*v*) skimmed milk for 24 h. **(B,C)** Motility phenotypes of *P. aeruginosa* PAO1 and its isogenic QS LasR^Q45stop^ strains. Image showing the comparative zone of twitching **(B)** and swarming motility **(C)** of PAO1 and its isogenic QS LasR^Q45stop^ strains. All data shown are the average values of three independent experiments ± SD. **(D,E)** Cultures of *P. aeruginosa* PAO1, a LasR^Q45stop^ mutant, and the evolved LasR^Q45stop^ variants, grown in LP medium for 24 h **(D)** and casein broth for 48 h **(E)**. The culture is visibly blue as a result of pyocyanin overproduction. Three biological replicates were performed. All data shown are the average values of three independent experiments ± SD. Two-tailed *p*-values were determined using unpaired *t*-tests. Statistical significance: ^*^p < 0.05, ^**^p < 0.01, ^***^p < 0.001, and ^****^p < 0.0001; ns, no significant difference (*p* ≥ 0.05). Scale bar = 10 mm.

LasR mutations are prevalent in *P. aeruginosa* isolated from chronic infections (Feltner et al., 2016). Interestingly, many LasR-null clinical isolates partially restored the QS regulon over extended infections (Feltner et al., 2016). This suggests that post-LasR-deficient evolution might confer diverse benefits. We were intrigued by the evolvability and evolutionary trajectory of the high-frequency mutant LasR^Q45stop^ in minimal medium with casein as the exclusive carbon and energy source. A competitive assay between the LasR^Q45stop^ variant and WT was conducted as described above (Supplementary Figure S2C). LasR^Q45stop^ variants were isolated using LB Gm-resistant plates, and their protease activity was assessed on skim milk agar plates (Supplementary Figure S2D). Variants exhibiting notable protease alterations were selected for further analysis (Supplementary Figure S2D). We identified four protease-altered LasR^Q45stop^ variants from these evolutionary experiments. Variants LasR^Q45stop^-1, LasR^Q45stop^-2, and LasR^Q45stop^-4 demonstrated reduced protease activity relative to the parental strain LasR^Q45stop^. In contrast, LasR^Q45stop^-3 exhibited increased protease activity, approaching that of the wild-type strain (Figure 2A). We also evaluated the influence of these four isolates on virulence-associated traits, such as cell motility and pyocyanin production. Consequently, the twitching motility of LasR^Q45stop^-1, LasR^Q45stop^-2, and LasR^Q45stop^-4 was diminished compared to the parental strain LasR^Q45stop^ (Figure 2B). Remarkably, LasR^Q45stop^-3 amplified swarming motility, while LasR^Q45stop^-1, LasR^Q45stop^-2, and LasR^Q45stop^-4 did not exhibit significant deviations from their parental strain LasR^Q45stop^ (Figure 2C). Additionally, pyocyanin production was elevated in LasR^Q45stop^-3 (Figure 2D). Of note, LasR^Q45stop^-4 maintained functional pyocyanin production, independent of its protease activity phenotype (Figures 2A,D).

### Mutations in *mexT* and *xcpA* are associated with the post-*lasR*-deficient evolution phenotype

To uncover the genetic variations responsible for the observed differences in virulence-associated phenotypes, we conducted a genome-wide association study among the parent LasR^Q45stop^ and four protease-altered mutants (LasR^Q45stop^-1, LasR^Q45stop^-2, LasR^Q45stop^-3, and LasR^Q45stop^-4). This analysis identified three genes: *mexT* (PA2492), *xcpA* (PA4528), and *psdR* (PA4499) (Table 1). The single base indel or in-frame deletion in the *xcpA* gene (Table 1) was found in LasR^Q45stop^-1, LasR^Q45stop^-2, and LasR^Q45stop^-4. Conversely, a missense variant in the *mexT* gene was identified in LasR^Q45stop^-3 (Table 1). The LasR^Q45stop^-4 subline displayed additional structural variations (SVs), culminating in the deletion of the entire *mexT* gene and adjacent genes spanning from PA2259 to PA2544. This range is part of the 38.3 kb deletion observed in LasR^Q45stop^-4. Furthermore, we noted that all four sequenced isolates carried a nonsynonymous mutation in *psdR*, aligning with prior research that identified frequent *psdR* mutations in casein evolution experiments (Asfahl et al., 2015; Kostylev et al., 2019). Nevertheless, *psdR* has been demonstrated to enhance growth on casein independently of QS (Asfahl et al., 2015). We thus infer that *MexT* and *XcpA* are instrumental in the observed virulence-associated phenotypic variations in the evolved isolates.

**Table 1:** Summary of *xcpA* and *mexT* variants found in in protease-modified variants of PAO1 LasR^Q45stop^ isolates that emerged during growth in caseinate.

Strains	*mexT* genotype	*xcpA* genotype	Variation	Other affected genes
**LasR**^Q45stop^**variants**				
LasR^Q45stop^-1	Normal	D (63)	Frame-shift deletion	*psdR*
LasR^Q45stop^-2	Normal	D (607–648)	Truncation	*psdR*
LasR^Q45stop^-3	A1044G	Normal	Base substitution	*psdR*
LasR^Q45stop^-4	2,489,242–2,871,766	D (607–648)	SV, Truncation	*psdR*, *PA2254-PA4499*

Variants LasR^Q45stop^-1 to LasR^Q45stop^-4 were identified in LasR^Q45stop^ protease-modified isolates that emerged during growth in caseinate and were initially inoculated with the WT only. “D” denotes base deletion; numbers specify the nucleotide position of the mutation.

**Figure 3:**
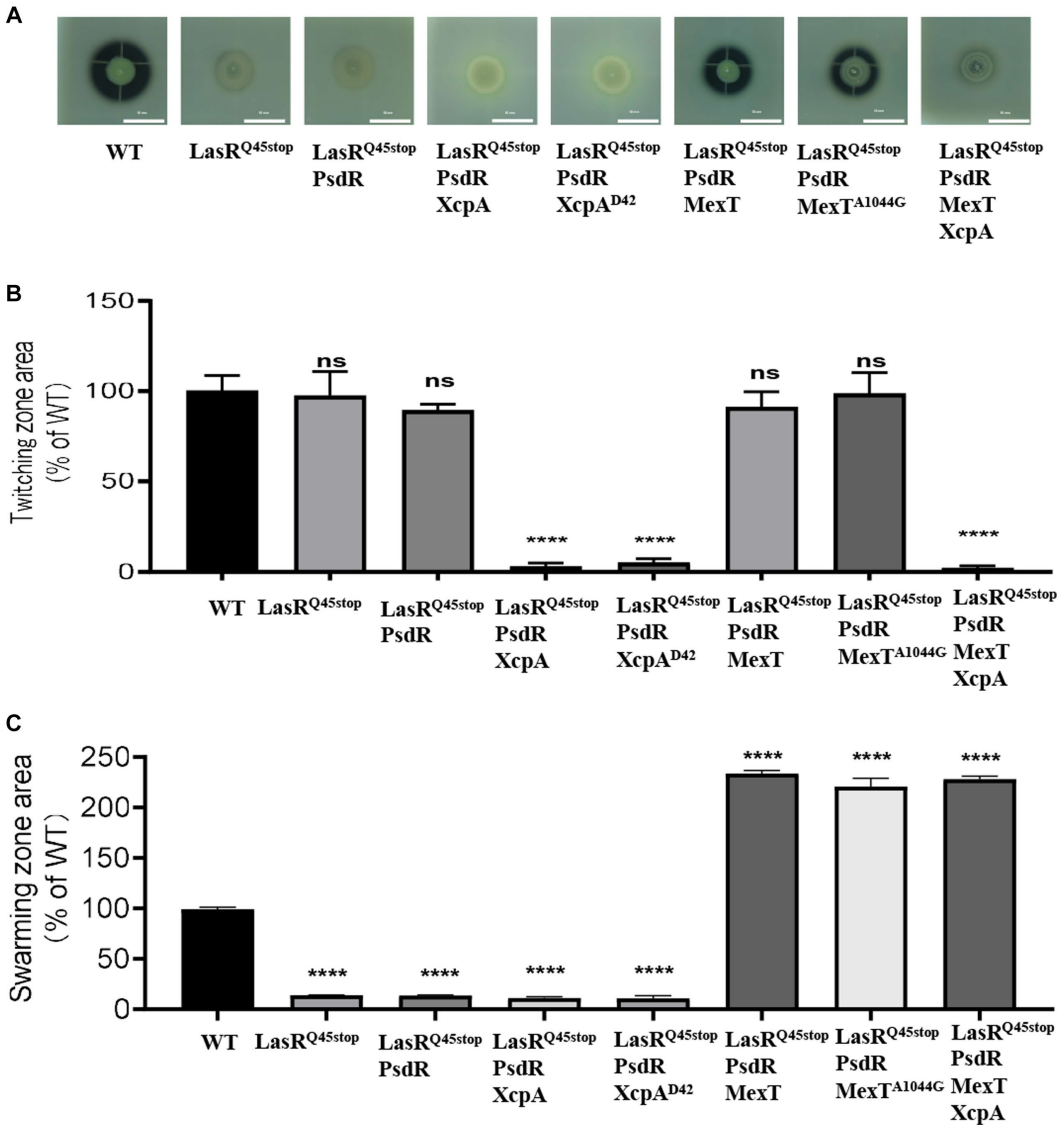
*mexT* and *xcpA* was related to the phenotype changes. **(A)** The influence of PsdR, MexT, and XcpA mutations on protease production in the *P. aeruginosa* LasR^Q45stop^ mutant. The images are of colonies after about 24 h of incubation in milk agar plate. The influence of PsdR, MexT, and XcpA mutations on twitching **(B)** and swarming **(C)** motility phenotypes in the *P. aeruginosa* LasR^Q45stop^ mutant. Three biological replicates were performed. All data shown are the average values of three independent experiments ± SD. Two-tailed *p*-values were determined using unpaired *t*-tests. Statistical significance: ^*^p < 0.05, ^**^p < 0.01, ^***^p < 0.001, and ^****^p < 0.0001; ns, no significant difference (*p* ≥ 0.05). Scale bar = 10 mm.

To substantiate this, we engineered MexT-and XcpA-related mutants using LasR^Q45stop^-PsdR as the progenitor strain. Our results show that LasR^Q45stop^-PsdR-MexT and LasR^Q45stop^-PsdR-MexT^A1044G^ mutants exhibited markedly elevated pyocyanin and elastase production (Figure 3), as well as augmented swarming motility (Figure 3). Deletion of *xcpA* from both LasR^Q45stop^-PsdR and LasR^Q45stop^-PsdR-MexT mutants led to reduced QS-dependent elastase phenotypes and twitching motility (Figure 3). Alterations in *mexT* have been highlighted as pivotal in reshaping the QS circuits and the fitness of *P. aeruginosa* PAO1 (Kostylev et al., 2019). *XcpA* is a prepilin peptidase that is part of the *Xcp* type II secretion system (T2SS) in *P. aeruginosa*. Furthermore, the *Xcp* T2SS is governed by the QS pathway, which *P. aeruginosa* utilizes for secreting major virulence factors, including LasA and LasB elastases (Li and Lee, 2019). Therefore, we conclude that the *xcpA* and *mexT* mutations underpin the evolved phenotype, at least under laboratory conditions.

### The *xcpA* mutant gains a fitness advantage in caseinate

**Figure 4:**
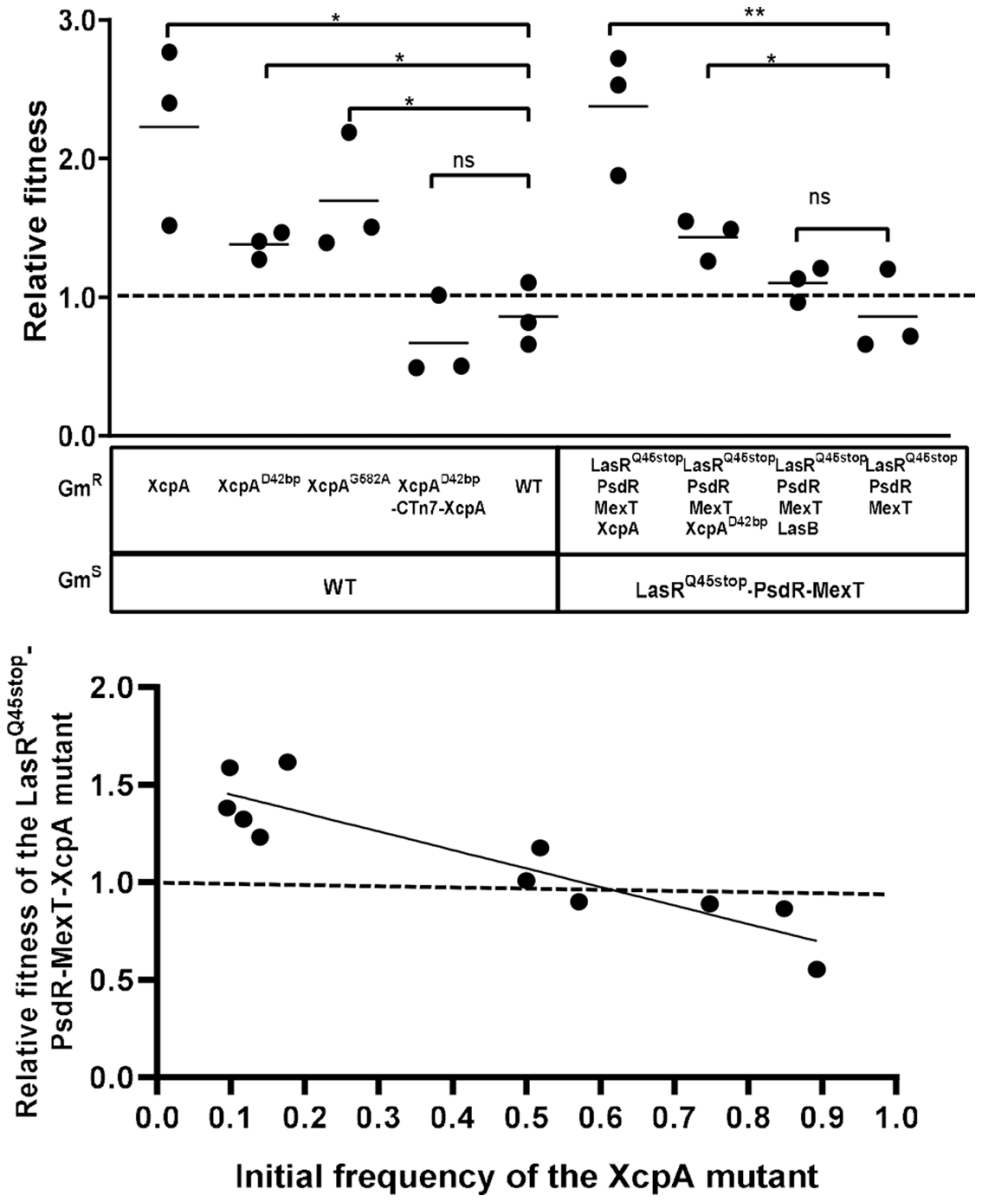
Growth and competition of the MexT mutant, the XcpA mutant, the LasB mutant and the parent strain. **(A)** Competition experiments showing the competitive indexes of competitor strains against the parent strain (WT or LasR^Q45stop^-PsdR-MexT) after 24 h in PM casein broth. Each symbol represents the outcome of an individual experiment; the solid lines represent means for each group. The starting percentage of the competitors was 10%. The LasB mutant was not fit than the parent, and the XcpA mutant was more fit than the parent. **(B)** The relative fitness of a XcpA mutant exhibits a negative frequency dependence. Data are from 24 h casein broth cultures. The outcomes above the dashed line indicate the competitor had a fitness advantage and below indicated the parent LasR^Q45stop^-PsdR-MexT had a fitness advantage. All data shown are the average values of three independent experiments ± SD. Two-tailed *p*-values were determined using unpaired *t*-tests. Statistical significance: ^*^p < 0.05 and ^**^p < 0.01; ns, no significant difference (*p* ≥ 0.05).

Mutants devoid of the metabolic cost of QS could possess a negative frequency-dependent fitness advantage over the parental strain when co-cultured in an environment where QS function is essential for growth. In previous research, LasR-MexT mutants exhibited a competitive advantage over the parental LasR mutant (Kostylev et al., 2019). Similarly, we postulated that XcpA and LasB mutants might also harbor a competitive edge. We co-cultured each identified mutant with the parental strain, starting at a frequency of 10%, to evaluate their relative fitness in PM-caseinate. Distinguished mutants were marked as gentamicin-resistant (Gm^R^). Relative fitness was ascertained by taking the ratio of mutant-to-parent bacteria at the endpoint and dividing it by the initial mutant-to-parent bacteria ratio (Chen et al., 2019). A control was set up with unlabeled and labeled parental strains, yielding a relative fitness of 1 (Figure 4A). Conversely, the relative fitness of the XcpA mutant, when pitted against LasR^Q45stop^-PsdR-MexT or WT, exceeded 1, indicating a survival advantage in the XcpA mutant. However, the LasB mutant, which only lacked elastase production, exhibited a fitness comparable to the LasR^Q45stop^-PsdR-MexT parent strain (Figure 4A). Additionally, varying proportions of XcpA mutants were co-cultured with their parent strains in PM-caseinate for 48 h to determine their frequency-dependent fitness. When XcpA mutants were more abundant relative to LasR^Q45stop^-PsdR-MexT in the culture, their relative fitness was less than 1. However, when LasR^Q45stop^-PsdR-MexT bacteria predominated over the mutant in the starting inoculum, the relative fitness for XcpA exceeded 1 (Figure 4B). The relative fitness of the mutants declines as the mutant subpopulation expands (Diggle et al., 2007). These observations underscore that XcpA mutants can effectively invade their ancestral populations under conditions that require QS.

## Discussion

*P. aeruginosa*, a well-known human pathogen, is particularly adept at infecting immunocompromised individuals, leading to severe infections (Moradali et al., 2017). Early work highlighted the complexity of interconnected quorum sensing (QS) regulatory circuits in controlling virulence phenotypes and adapting to complex environments (Willcox et al., 2008). The QS transcription factor LasR sits atop this hierarchy, regulating the expression of the RhlIR and PQS pathways (Cruz et al., 2020). LasR mutations have been shown to be prevalent in *P. aeruginosa* during both *in vitro* and *in vivo* evolution. Analysis of the associated *lasR* sequences from CF isolates (Feltner et al., 2016) indicated that LasR^Q45stop^ is a high-frequency mutant (Supplementary Figure S1 and Supplementary Table S2). QS-regulated biological phenotypic assessments were diminished in LasR^Q45stop^ mutants. However, these assessments were restored to wild-type levels upon the addition of functional LasR (Figure 1). This suggests that LasR^Q45stop^ might function as a QS antagonist, inhibiting *P. aeruginosa* infection. Paradoxically, inactivating LasR mutations are frequently detected in isolates from chronic CF infections, suggesting an evolutionary selection favoring LasR mutants (Cruz et al., 2020). Recent research indicates that defects in the LasIR system can result in the evolution of MexT and PqsR mutations under laboratory conditions, leading to reactivation of *P. aeruginosa* QS regulatory circuits through rewiring (Chen et al., 2019; Kostylev et al., 2019). Phenotypic and genotypic microevolution result from niche adaptation to the CF lung environment and competition among different microbiomes. Micro-evolutionary diversity changes dynamically over time, complicating the accurate diagnosis and treatment of *P. aeruginosa* infections (Hwang et al., 2021).

The inherent genetic diversity of *P. aeruginosa* has been elucidated due to its suitability for adaptive laboratory evolution experiments. We identified LasR^Q45stop^ evolutionary trajectories caused by acquiring stable mutant phenotypes, leading to rapid adaptation in stressful and fluctuating environments. Our findings substantiate the implications of LasR^Q45stop^ microevolution. We employed a combination of molecular biology and bioinformatics analyses to study the evolution of LasR^Q45stop^ cessation. We demonstrated that LasR^Q45stop^ promotes an increased frequency of *psdR*, *mexT*, and *xcpA* mutations when co-cultured in our caseinate broth (Table 1). The *psdR* mutation enhances individual fitness by alleviating repression of intracellular metabolism, limiting growth. This mutation has been frequently observed in evolution experiments involving casein (Asfahl et al., 2015; Kostylev et al., 2019). Even with *psdR* deletion in both cooperators and cheaters, the pattern of invasion by the LasR mutant remains largely unaffected (Feng et al., 2019). The *mexT* mutation isolates have been identified both in the lab and clinical settings (Liu et al., 2022), while *xcpA* mutations frequently appear in environmental isolates (Supplementary Table S3). Moreover, our data highlight the role of MexT and XcpA in the adaptation of *P. aeruginosa*. Microbial cells exhibit substantial adaptation to environmental stress (Hogardt and Heesemann, 2010). Certain pivotal mutations in the genome can significantly bolster bacterial populations in securing a competitive edge in hostile environments (Feng et al., 2019).

For survival in the environment and adaptation to hosts for nourishment and pathogenicity, *P. aeruginosa* deploys a diverse array of virulence factors (Vidaillac and Chotirmall, 2021). Among these, the T2SS-peptidase XcpA is involved in the secretion of guanylate cyclase ExoA, proteases LasA/B, and several other factors, many of which have been earmarked as potential therapeutic targets (Swietnicki et al., 2019). Importantly, the *lasB* gene encodes elastase, a pivotal virulence component of *P. aeruginosa*. Its synthesis is orchestrated by the QS system, while its secretion modus operandi is overseen by the T2SS, also referred to as the general secretory pathway (GSP) (Filloux, 2004). The 12 identified Gsp components of the *P. aeruginosa* machinery were initially labeled as XcpA and XcpP to-Z and later renamed as XcpA_O_ and XcpP_C_ to-Z_M_, aligning with other GSP system nomenclature (Bleves et al., 2010). XcpA, recognized as a prepilin peptidase, was initially discovered and described in *P. aeruginosa*. It is believed to excise leader peptides endopeptidically, methylate the N-terminal amino acid residue, and cleave Xcp pre-pseudopilin substrates (Filloux, 2011). Few studies have explored the essential biological activities of prepilin peptidase in bacteria (LaPointe and Taylor, 2000). Tn insertions in the *xcpA* genes of *P. aeruginosa* strain PA14 manifested positive selection during fitness for gastrointestinal (GI) colonization but predominantly displayed attenuated fitness for systemic dissemination amidst neutropenia. This observation stemmed from a reliable mouse model mirroring the trajectory of human infections in cancer and bone marrow transplant patients (Koh et al., 2005). In *Methanococcus voltae*, FlaK operates as a preflagellin peptidase; membranes of the *flaK* mutant exhibited inability to process preflagellin *in vitro* (Bardy and Jarrell, 2003). In *Bacillus subtilis*, ComC functions as a prepilin peptidase; its inactivation results in the accumulation of pre-ComGC, likely due to the unprocessed protein’s inability to translocate outside the membrane (Chung and Dubnau, 1995). The significance of XcpA, a class of prepilin peptidase, in processing secretion systems has been documented. Pathogenic bacteria and environmental entities could capitalize on the extracellular dispatch of an array of toxins, lipases, and enzymes adept at breaking down complex materials, mediated through the secretory system (Korotkov and Sandkvist, 2019).

This study sheds light on the role of XcpA in *P. aeruginosa* evolutionary paths. Our findings indicate that the XcpA mutation confers a fitness advantage. When mutant XcpA is present in PsdR-LasR^Q45stop^-MexT, it reduces exoprotein secretion and enhances individual fitness. Although the physiological value of *xcpA*-associated microevolution is not fully detailed in this study, we delve into its role in fitness and intraspecific competition in casein broth. Our evaluation scope is limited to studying individual LasR variants and their evolution with casein proteins under laboratory conditions. Notably, pathogenic adaptation mutations are not consistent across studies, suggesting diverse evolutionary paths of pathogenic adaptation in *P. aeruginosa*. Still, our findings pave the way for understanding LasR’s evolutionary paths under lab conditions and potential further studies on XcpA in population evolution.

## Data availability statement

Genomic sequences of LasR^Q45stop^-1, LasR^Q45stop^-2, LasR^Q45stop^-3, and LasR^Q45stop^-4 have been deposited at NCBI (BioProject PRJNA967237).

## Author contributions

MC: Data curation, Investigation, Methodology, Writing – original draft, Writing – review & editing. RC: Methodology, Resources, Supervision, Writing – review & editing. LL: Methodology, Project administration, Resources, Supervision, Writing – original draft, Writing – review & editing.
