# Supplementary material for: T2SS-peptidase XcpA associated with LasR evolutional phenotypic variations provides a fitness advantage to Pseudomonas aeruginosa PAO1
Source: Front Microbiol. 2023 Oct 26;14:1256785. doi: 10.3389/fmicb.2023.1256785 (PMC10637944; doi:10.3389/fmicb.2023.1256785)

SI Appendix

SUPPLEMENTAL MATERIAL for:

T2SS-peptidase XcpA associated with LasR evolutional phenotypic variations provides a fitness advantage to *Pseudomonas aeruginosa* PAO1

Table S1. Strains, plasmids, and primers used in this study.

Strain or plasmid	Relevant characteristic(s) [a]	Source
P. aeruginosa		
PAO1	Wildtype	Lab collection
LasR	PAO1 containing an unmarked, in-frame *lasR* deletion	This study
LasR^{Q45stop}	Homologous mutation of *lasR*^{C133T} in laboratory *Pseudomonas aeruginosa* strain PAO1	This study
C-LasR^{Q45stop}	Complement LasR in LasR^{Q45stop}	This study
PsdR	PAO1 containing an unmarked, in-frame *psdR* deletion	This study
XcpA	PAO1 containing an unmarked, in-frame *xcpA* deletion	This study
XcpA^{G582A}	Homologous mutation of *xcpA*^{G582A} in laboratory *Pseudomonas aeruginosa* strain PAO1	This study
XcpA^{D42bp}	PAO1 containing an unmarked, contains an in-frame *xcpA*$^{D(607\text{-}648)}$ deletion	This study
XcpA^{D42bp}-CTn7-XcpA	Complement XcpA in XcpA^{D42bp}	This study
LasR^{Q45stop}-PsdR	Derived from LasR^{Q45stop}; contains an in-frame *psdR* deletion	This study
LasR^{Q45stop}-PsdR-MexT	Derived from LasR^{Q45stop}-PsdR; contains an in-frame *mexT* deletion	This study
LasR^{Q45stop}-PsdR-MexT^{A1044G}	Derived from LasR^{Q45stop}-PsdR; contains an base replace of *mexT*^{A1044G}	This study
LasR^{Q45stop}-PsdR-MexT-XcpA	Derived from *lasR*^{Q45stop}-PsdR-MexT; contains an in-frame *xcpA* deletion	This study

lasR^{Q45stop}-PsdR-MexT-XcpA^{D42bp}	Derived from LasR^{Q45stop}-PsdR-MexT; contains an in-frame *xcpA*$^{D(607\text{-}648)}$ deletion	This study
LasR^{Q45stop}-PsdR-MexT-LasB	Derived from LasR^{Q45stop}-PsdR-MexT; contains an in-frame *lasB* deletion	This study
**LasR^{Q45stop} mutant variants**		
LasR^{Q45stop}-1	Isolated from LasR^{Q45stop} evolution in 1% casein	This study
LasR^{Q45stop}-2	Isolated from *lasR*^{Q45stop} evolution in 1% casein	This study
LasR^{Q45stop}-3	Isolated from LasR^{Q45stop} evolution in 1% casein	This study
LasR^{Q45stop}-4	Isolated from LasR^{Q45stop} evolution in 1% casein	This study
E. coli		
DH5α	*supE44*, *hsdR17*, *recA1*, *endA1*, *gyrA96*, *thi-1*, *relA1*	Novagen
pRK2013	ori colE1, RK2 derivative, Km^{R} mob^{+} tra^{+}	Lab collection
**Plasmids**		
pProbe-GT-P$_{lasI}$*gfp*	pVS1/p15a vector with *gfp* under the control of P$_{lasI}$	Lab collection
pEXG2	Allelic exchange vector with pBR origin, *sacB*, Gm^{R}	Lab collection
pEXG2-*lasR*	pEXG2 containing sequences for *lasR* knockout	This study
pEXG2-*lasR*^{Q45stop}	pEXG2 containing sequences for *lasR*^{Q45stop} knockout	This study
pEXG2-*psdR*	pEXG2 containing sequences for *psdR* knockout	This study
pEXG2-*mexT*	pEXG2 containing sequences for *mexT* knockout	This study
pEXG2-*mexT*^{A1044G}	pEXG2 containing sequences for *mexT*^{A1044G} knockout	This study
pEXG2-*xcpA*^{D42bp}	pEXG2 containing sequences for *xcpA*^{D42bp} knockout	This study
pEXG2-*xcpA*	pEXG2 containing sequences for *xcpA* knockout	This study
pEXG2-*lasB*	pEXG2 containing sequences for *lasB* knockout	This study
pUC18-mini-Tn7T-Gm	Gm^{R} on mini-Tn7T; for gene insertion in Gm^{S} bacteria	Lab collection
pUC18-mini-Tn7T-Gm-*lasR*	Derived from pUC18-mini-Tn7T-Gm ; used for the integration of *lasR* at the *att* site	This study
pUC18-mini-Tn7T-Gm-*xcpA*	Derived from pUC18-mini-Tn7T-Gm ; used for the integration of *xcpA* at the *att* site	This study

pFLP2	Source of Flp recombinase, Amp^R/Cb^R	Lab collection
pTNS2	Source of transposase, Amp^R	Lab collection
**Primers**		
lasR-UP-F	5'– gcataaatgtaaagcaagcttCCGAACTGGAAAAGTGGCTATG –3'	
lasR-UP-R	5'– gcaagaAGCGCTACGTTCTTCTTAAACTATTAA –3'	
lasR-DN-F	5'– aagaacgtagcgctTCTTGCCTCTCAGGTCGGC –3'	
lasR-DN-R	5'– cgagctcgagcccggggatccTAACCATCGATTTCCATCTCGTC –3'	
KO-*lasR*-detect-F	5'– GTGGGCTGACTGGACATCTT –3'	
KO-*lasR*-detect-R	5'– TCAGAGCAATGGCTTCACAC –3'	
lasR^{Q45stop}-UP-F	5'– gcataaatgtaaagcaagcttCCGAACTGGAAAAGTGGCTATG –3'	
lasR^{Q45stop}-UP-R	5'– ttctcgtagtcctaGCTGTCCTTAGGCAACAGGCC –3'	
lasR^{Q45stop}-DN-F	5'– taaggacagcTAGGACTACGAGAACGCCTTCAT –3'	
lasR^{Q45stop}-DN-R	5'– CgagctcgagcccggggatccCAGTCGTTTCGAGAATGGCG –3'	
psdR-UP-F	5'– gcataaatgtaaagcaagcttTTCCTCCCAGGTGTGGATGG –3'	
psdR-UP-R	5'– cTGGGGAGACTCTTGTTGAAAGG –3'	
psdR-DN-F	5'– ttcaacaagagtctccccaGCCAGTCCGTAGCGCACC –3'	
psdR-DN-R	5'– cgagctcgagcccggggatccAGCTTTCCGCCAGGGCCG –3'	
KO-*psdR*-detect-F	5'– CCATGCTCGCCCATCCA –3'	
KO-*psdR*-detect-R	5'– GTCCGTTTGTTGTTGGTCG –3'	
mexT-UP-F	5'– gcataaatgtaaagcaagcttCGCCGGCCAGTTCGAAGC –3'	
mexT-UP-R	5'– cagGCTGCGTTCGTGCATCAGG –3'	
mexT-DN-F	5'– tgatgcacgaacgcagcCTGGCGGTGCCGCAGTTC –3'	
mexT-DN-R	5'– cgagctcgagcccggggatccGGAGAAGTGGGATGACTGTTCC –3'	
KO-*mexT*-detect-F	5'– CTGCTCCGGGGCCAGGTTCT –3'	
KO-*mexT*-detect-R	5'– ATGCCTTGGGTGGTTTCC –3'	

xcpA-UP-F	5’– cgagctcgagcccggggatccTGCATAAACGCTCGAAAAAATT –3’
xcpA-UP-R	5’– atccagggttgggCAGTTATCCGACGACGTTGCC –3’
xcpA-DN-F	5’– ataactgCCCAACCCTGGATACTAGGCC –3’
xcpA-DN-R	5’– atgtaaagcaagcttctgcagCAAGCGCTCGTCGCGCCG –3’
KO-*xcpA*-detect-F	5’– GACCTTGTCCACGACCTTC –3’
KO-*xcpA*-detect-R	5’– GACGCCGAGGGAGATGAAG –3’
xcpA^{D42bp}-UP-F	5’– gcataaatgtaaagcaagcttTGCCGAAGATGATGGAGCG –3’
xcpA^{D42bp}-UP-R	5’– agCAGCTTGAACAGCCAGAACACC –3’
xcpA^{D42bp}-DN-F	5’– ttctggctgttcaagctgCTGGCCATGCTCGGTGCC –3’
xcpA^{D42bp}-DN-R	5’– cgagctcgagcccggggatccCAGCGGGTGCAGCAATTG –3’
xcpA^{D42bp}-UP-R1	5’– CAGCTTGAACAGCCAGAACACC –3’
xcpA^{D42bp}-DN-F1	5’– CTGGCCATGCTCGGTGCCT –3’
lasB-UP-F	5’– atgtaaagcaagcttctgcagGCAGCAGCGGATCGTCGG –3’
lasB-UP-R	5’– TTGTAGTTGCTGCAGCCTTGCTGGGGAGTTTGG –3’
lasB-DN-F	5’– CAGCAAGGCTGCAGCAACTACAACAGCGGC –3’
lasB-DN-R	5’– cgagctcgagcccggggatccGCCAGGTACTCGCCTTGCG –3’
KO-*lasB*-detect-F	5’– TACAAGCTCGACGTCAACGAA –3’
KO-*lasB*-detect-R	ACCTGAACTTTAGACCGGG –3’
pGEX2-F	5’– CGAGCCGGAAGCATAAATGT –3’
pGEX2-R	5’– TCCGCGTTTCCAGACTTTAC –3’
lasR-Compl-F1	5’– CCGCCGCGAGGTCGCCCCC –3’
lasR-Compl-R1	5’– TCAGAGAGTAATAAGACCCAAATT –3’
Tn7-P*lasR*-F2	5’– CGATCATGCATGAGCTCCGCCGCGAGGTCGCCCCCT –3’
Tn7-*lasR*-R2	5’– GGTACCGGGCCCAAGCTTTCAGAGAGTAATAAGACCCA AAT –3’
xcpA-Compl-F	5’– gaaattaagcATGCCCCTCCTCGACTACCT –3’
xcpA-Compl-R	5’– GGGCCCAAGCTTTCATTTGAATCCGGCGAATTGC –3’
Tn7-Prrnb-F	5’– AATTCGATCATGCATGAGCTCgttgcgcggtcagaaaattatt –3’
Prrnb-*xcpA*-R	5’– AGGAGGGGCATgcttaatttctcctctttaat –3’

a Resistant to ampicillin (Amp^R), carbenicillin (Cb^R),kanamyceticus (Km^R),or gentamicin (Gm^R)

Table S2. LasR^{Q45stop} is a high-frequency mutation site of the *lasR* variants

lasR variants/ rate	Case number of *lasR* varian	Case number of patients
LasR^{Q45stop}	50	21
Total LasR variants	441	205
Rate	11.3%	10.2%

Table S3. *xcpA* mutations in the environment

BioSample	*xcpA* mutation	Source	Date
SAMN09389275	C4A, Pro2Thr; C52G, Thr18Ala	hospital	2019
SAMN11126830	C4A, Pro2Thr; C90T, nonsense mutation	potato	2019
SAMN11087507	C4A, Pro2Thr; G141A, nonsense mutation; T798G, nonsense mutation	Cystic Fibrosis lung sputum	2019
SAMN15195942	C4A, Pro2Thr; G141A, nonsense mutation; C672T, nonsense mutation;	hospital	2022
SAMN23605131	C4A, Pro2Thr; C672T, nonsense mutation	sputum	2022
SAMN23553085	C4A, Pro2Thr; C780T, nonsense mutation; T798G, nonsense mutation	soil	2021
SAMN04351367	C4A, Pro2Thr; G141A, nonsense mutation	Blood culture of patient	2016
SAMN02383553	C4A, Pro2Thr; C52G, Thr18Ala; C63T, nonsense mutation	Patient (nose)	2016
SAMN05729608	C4A, Pro2Thr; C90T, nonsense mutation	wastewater	2016
SAMN10503341	C4A, Pro2Thr; C52G,Thr18Ala	urine	2018
SAMN08101545	C90T, nonsense mutation; T798G, nonsense mutation	urine	2017
SAMN15196026	C4A, Pro2Thr; C780T, nonsense mutation; T798G, nonsense mutation	respiratory	2022

FIGURE S1 Summary of the *lasR* variants in the EPIC *P. aeruginosa* collection based on comparison to *P. aeruginosa* PAO1 *lasR* sequence. Red columns indicate the case number of *lasR* variants mutation site. Black lines indicate the case number of patients whom *lasR* variants isolated from.

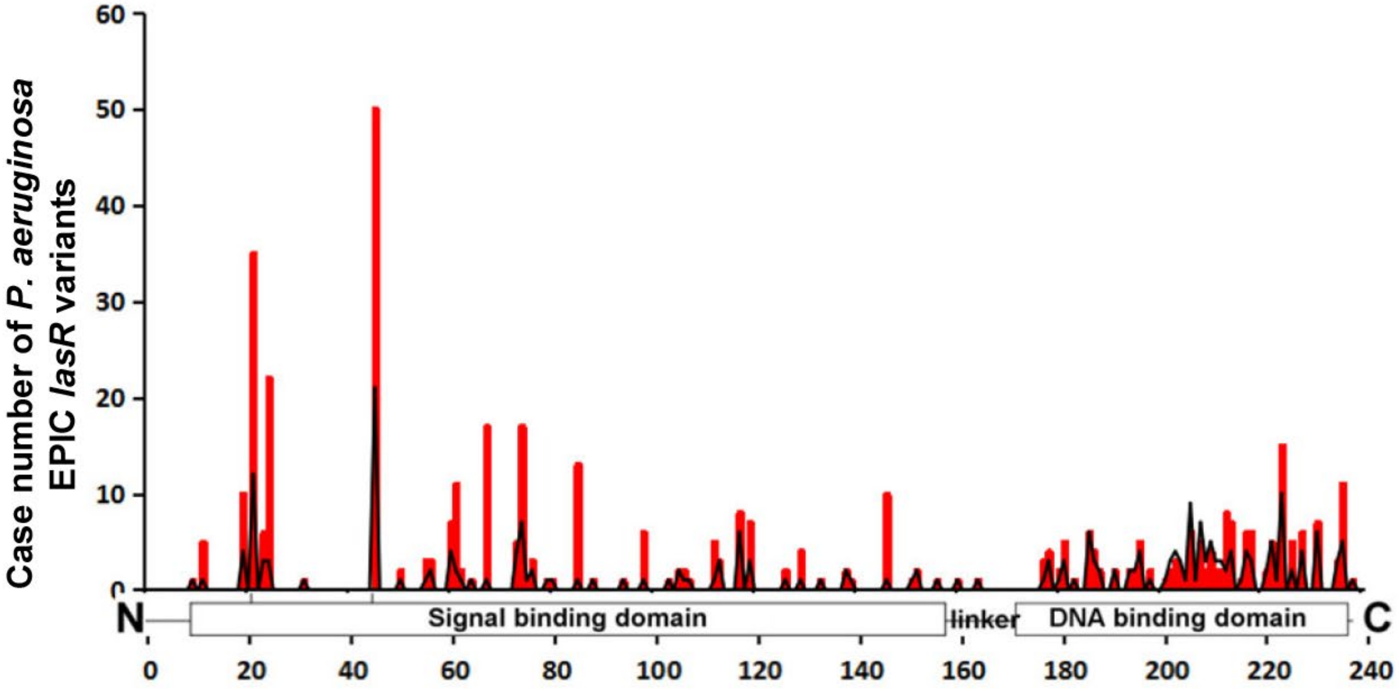

FIGURE S2 LasR^{Q45stop} mutant protease-change variants isolated from co-cultures of the WT. (A) The comparison of WT and WT co-cultured with LasR variants social evolution experiments. The crush day of WT in casein broth(closed circles); The crush day of WT co-cultured with 1% LasR in casein broth (closed triangles); The crush day of WT co-cultured with 1% LasR^{Q45stop} in casein broth (closed squares). (B) The LasR^{Q45stop} variants had no significant effect on WT growth on LB-Mops medium. (C) Images of Experimental design. (D) A milk agar plate spotted with LasR^{Q45stop} variants. Image was taken after incubation at 37℃ for 14 h. The parent non-protease producer LasR^{Q45stop} and protease producer WT are at the top. All data shown are the average values of three independent experiments ± SD.

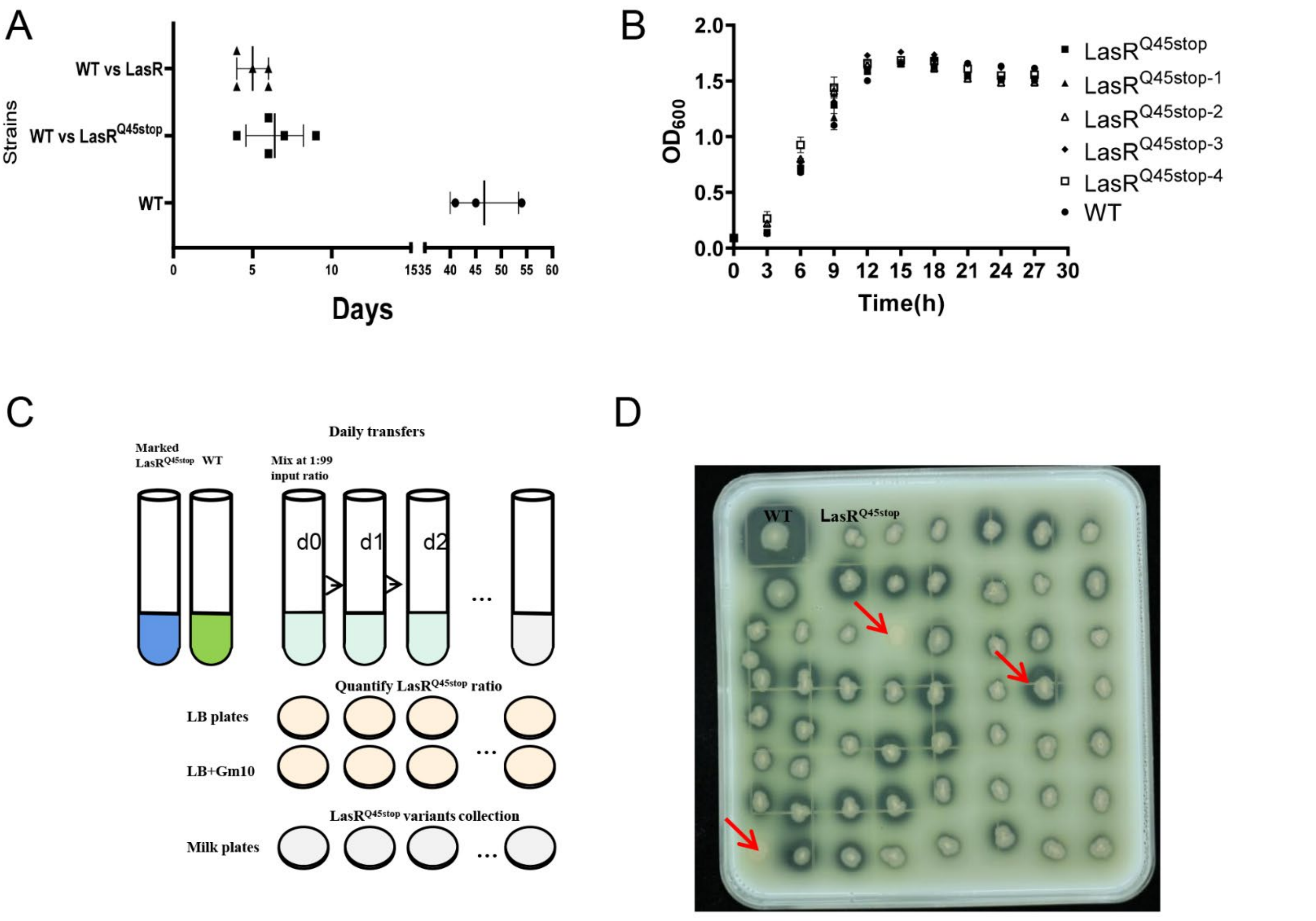

Supplement: Supplementary file 1 [file Data_Sheet_1.pdf]
